# Carotid intima-media thickness in spondyloarthritis patients

**DOI:** 10.1590/S1516-31802013000100020

**Published:** 2013-04-01

**Authors:** Thelma Larocca Skare, Guilherme Cortez Verceze, André Augusto de Oliveira, Sonia Perreto

**Affiliations:** I MD, PhD. Head of Rheumatology Unit, Hospital Universitário Evangélico de Curitiba, Paraná, Brazil.; II Student, School of Medicine, Faculdade Evangélica do Paraná (Fepar), Curitiba, Paraná, Brazil.; III MD. Cardiologist, Echocardiography Service, Hospital Universitário Evangélico de Curitiba, Curitiba, Paraná, Brazil.

**Keywords:** Spondylarthropathies, Carotid artery diseases, Inflammation, Atherosclerosis, Spondylitis, ankylosing, Espondiloartropatias, Doenças das artérias carótidas, Inflamação, Aterosclerose, Espondilite anquilosante

## Abstract

**CONTEXT AND OBJECTIVE:**

Accelerated atherosclerosis has become a major problem in rheumatic inflammatory disease. The aim here was to analyze carotid intima-media thickness (IMT) in spondyloarthritis (SpA) patients and correlate this with clinical parameters and inflammatory markers.

**DESIGN AND SETTING:**

Cross-sectional analytical study at Rheumatology Outpatient Clinic, Evangelical University Hospital, Curitiba.

**METHODS:**

IMTs (measured using Doppler ultrasonography) of 36 SpA patients were compared with controls. The IMT in SpA patients was associated with inflammatory markers, like erythrocyte sedimentation rate (ESR), C-reactive protein (CRP) and Bath Ankylosing Spondylitis Disease Activity Index (BASDAI); and with clinical parameters, like axial or peripheral involvement, dactylitis, HLA B27, uveitis occurrence, Bath Ankylosing Spondylitis Functional Index (BASFI) and lipid profile.

**RESULTS:**

The mean IMT in SpA patients was 0.72 ± 0.21 mm; in controls, 0.57 ± 0.13 mm (P = 0.0007). There were no associations with ESR, CRP, BASDAI or clinical data. In univariate analysis, greater IMT was seen in patients with longer disease duration (P = 0.014; Pearson R = 0.40; 95% confidence interval, CI = 0.06 to 0.65); higher triglycerides (P = 0.02; Spearman R = 0.37; 95% CI = 0.03 to 0.64); and older age (P = 0.0014; Pearson R 0.51; 95% CI = 0.21 to 0.72).

**CONCLUSION:**

SpA patients have a higher degree of subclinical atherosclerosis than in controls, thus supporting clinical evidence of increased cardiovascular risk in rheumatic patients.

## RESUMO

**CONTEXTO E OBJETIVO:**

A aterogênese acelerada tem se tornado um grande problema nas doenças reumáticas inflamatórias. O objetivo foi analisar a espessura da camada íntima-média (ECIM) da carótida em pacientes com espondiloartrite (ES) e relacioná-la com parâmetros clínicos e marcadores inflamatórios. TIPO DE ESTUDO E LOCAL: Estudo transversal analítico no Ambulatório de Reumatologia do Hospital Universitário Evangélico de Curitiba.

**MÉTODOS:**

A ECIM (medida por Doppler) de 36 pacientes com ES foi comparada com controles. A ECIM de pacientes com ES foi associada com marcadores inflamatórios, como velocidade de hemossedimentação (VHS), proteína C-reativa (PCR), *Bath Ankylosing Spondylitis Disease Activity Index* (BASDAI), e com parâmetros clínicos, como envolvimento axial e periférico, dactilite, HLA B27, ocorrência de uveíte, *Bath Ankylosing Spondylitis Functional Index* (BASFI) e perfil lipídico.

**RESULTADOS:**

A ECIM média em pacientes com ES foi de 0,72 ± 0,21 mm, enquanto nos controles foi de 0,57 ± 0,13 mm (P = 0,0007). Não se encontrou associação com VHS, PCR, BASDAI e dados clínicos. Em análise univariada, maior ECIM foi encontrado nos indivíduos com maior duração de doença (P = 0,014; R Pearson = 0,40; 95% intervalo de confiança, IC = 0,06 to 0,65), aumento nos triglicerídeos (P = 0,02; R Spearman = 0,37; 95% IC = 0,03 to 0,64) e maior idade (P = 0,0014; R Pearson 0,51; 95% IC = 0,21 to 0,72).

**CONCLUSÃO:**

Pacientes com ES têm maior grau de aterosclerose subclínica do que controles, dando suporte às evidências clínicas de aumento de risco cardiovascular em pacientes com doenças reumáticas.

## INTRODUCTION

Chronic inflammatory rheumatic diseases are considered nowadays to present a risk of cardiovascular events and increased cardiovascular mortality.^1^ Circulating mediators such as interleukin (IL)-6, tumor necrosis factor-alpha (TNF-alpha), C-reactive protein (CRP) and adhesion molecules secondary to rheumatic inflammatory processes contribute to all stages of atherosclerosis: from endothelial dysfunction to atheroma formation, plaque instability and thrombus development.^2^

The association between subclinical atherosclerosis and severity of inflammatory response has been clearly demonstrated in patients with rheumatoid arthritis^3^^,^^4^ and systemic lupus erythematosus.^4^ However, in relation to spondyloarthritis (SpA), studies have produced divergent results.^5^^,^^6^ In this latter group of diseases, inflammatory markers do not accurately reflect the underlying pathological events. It is well known that the association between CRP levels and inflammation is lower in SpA than in rheumatoid arthritis.^7^

SpA encompasses a group of diseases that include ankylosing spondylitis (AS), reactive arthritis (ReA), psoriatic arthritis (PsA), inflammatory bowel disease (IBD), related arthritis and undifferentiated SpA (uSpA).^8^ This wide clinical spectrum results from combinations of different features such as inflammatory spinal involvement, peripheral arthritis, enthesitis, dactylitis, uveitis, aortic incompetence and the presence of human leukocyte antigen (HLA)-B27.^9^

An analysis^10^ on mortality rate and causes of death among 398 patients with longstanding AS found that the group of patients that died had higher erythrocyte sedimentation rate (ESR). Gonzalez-Juanatey et al.^6^ found that carotid artery intima-media thickness (IMT) in AS patients without clinically evident cardiovascular disease was higher than in matched controls, although Chloe et al.^11^ could not confirm this finding.^11^

## OBJECTIVE

In the present study, we analyzed carotid artery IMT in a cohort of local patients with SpA, comparing them with patients without rheumatic inflammatory disease, in order to investigate the presence of subclinical atherosclerosis. We further analyzed the association of clinical parameters and inflammatory activity in SpA with cardiovascular risks.

## METHODS

This was a cross-sectional analytical study that was approved by the local Research Ethics Committee, and all participants signed a consent statement. We included 72 patients: 36 SpA patients who fulfilled the ESSG (European Spondyloarthropathy Study Group)^12^ classification criteria for diagnosing SpA and 36 controls. The sample of SpA patients was formed by all the patients with a SpA diagnosis who were seen at our Outpatient Clinic from January 2011 to July 2011 (number estimated to be 55 patients) and who agreed to participate in the study.

The control group was selected among patients who sought the hospital for cataract and lower-leg varicose vein surgery without any known inflammatory disease. We excluded patients who had already experienced cardiovascular disease, heart failure, cerebrovascular events, peripheral arterial disease or renal insufficiency (creatinine above 1.2 mg/dl), or who were on anticoagulants.

The patients and controls were interviewed to obtain demographic data and data on associated diseases and tobacco and medication use. Patients were considered to have hypertension if their blood pressure was greater than 150/90 mm on at least two occasions.^13^ Information relating to diabetes mellitus was accessed through the patient’s history. For all patients and controls, cholesterol, triglyceride, low-density lipoprotein (LDL) cholesterol and high-density lipoprotein (HDL) cholesterol levels were determined after fasting overnight, by means of the enzymatic colorimetric method.

The SpA patients also gave responses to the Bath Ankylosing Spondylitis Disease Activity Index (BASDAI)^14^ and Bath Ankylosing Spondylitis Functional Index (BASFI)^15^ questionnaires. Their ESR was determined using the Westergreen method and CRP levels using immunoturbidimetry.

BASDAI is an instrument that measures disease activity in SpA, and this is done through measurement of five variables: spinal pain, peripheral joint pain, pain at enthesopathic sites, morning stiffness and fatigue, using a visual analogue scale. BASDAI values range from zero to 10, and values greater than 4 are considered to be suggestive of high levels of disease activity.^14^ BASFI is a functional index constructed through ten questions about daily activities that also score from zero to ten (where zero is the best possible performance and ten, the worst).^15^ Both BASDAI and BASFI have been validated for use in the Portuguese language.^16^

The SpA patients’ medical records were reviewed to obtain clinical data and ascertain whether HLA B27 was present. The clinical data gathered included: diagnosis of disease subtype, disease duration, uveitis, axial and/or peripheral arthropathy, enthesopathy, dactylitis and HLA B27 presence. AS was considered to be present if the patient fulfilled the New York modified criteria;^17^ PsA, if the patient fulfilled the Moll and Wright criteria;^18^ ReA, if asymmetric inflammatory oligoarthritis of the lower limbs was present in association with enthesopathy and/or inflammatory low back pain following enteric or urogenital infections;^19^ and enteropathic arthritis, when the patient presented inflammatory axial and/or peripheral joint involvement associated with confirmed inflammatory bowel disease (IBD) (Crohn’s disease or ulcerative colitis). Undifferentiated SpA was considered to be present when the patient presented with specific features of SpA without fulfilling the diagnostic criteria for one defined disease.^20^^,^^21^

A history of uveitis was considered to be present when diagnosed by an ophthalmologist. Peripheral arthritis was considered to be present when current or past synovitis was diagnosed by a doctor (in the 44-joint count).^22^ Assessment of axial involvement (sacroiliitis and/or spondylitis) was done through imaging the sacroiliac joints, lumbar and cervical spine by radiography and/or computed tomography scans.^22^ Enthesitis was considered to be present when the patient reported spontaneous pain or tenderness on examination at enthesis sites (according to the Maastricht Ankylosing Spondylitis Score).^22^ Dactylitis was considered to be present when current or past dactylitis was diagnosed by a doctor.^22^

Carotid IMT measurements were made on both sides using color Doppler equipment (ESAOTE, model MEGA CVX, with 7.5 MHz linear transducers). Transversal and longitudinal slices were imaged at the common carotid vessel, 3 cm below the bulbus. All tests were read by a single cardiologist who was blinded to clinical information. We considered that the patient had no thickening if the measurement on the intima-media complex was less than 0.8 mm. Thickening was present if the measurements were between 0.8 mm and 1.5 mm; and atheromatous plaques was present when the measurement was greater than 1.5 mm.^23^^,^^24^^,^^25^ For statistical calculations, we took the carotid IMT value that was greater, between the two sides.

The data obtained were studied using contingency and frequency tables. For association studies on nominal data, the chi-square and Fisher tests were used and for association studies on numerical data, the Mann-Whitney and unpaired t tests were used. For correlation studies, the Pearson and Spearman tests were applied. Calculations were done with the aid of the GraphPad Prism software, version 4.0. To further study the correlation of IMT with variables with P < 0.05, we performed multivariate analysis using the Medcalc software, version 12.1.3.0. The significance level used was 5%.

## RESULTS

Among the 36 SpA patients studied: 1/36 (2.7%) was ReA, 3/36 (8.33%) were PsA; 9/36 (25%) were USpA and 23/36 (63.88%) were AS. In the SpA group, the age range was from 19 to 74 years (mean 43.9 ± 10.97 years), the mean disease duration was 10.59 ± 10.57 years (range: from 1 to 48 years) and 47.2% were males and 52.7% were females. In this sample, we found that 90.62% had axial involvement, 53.12% entheseal involvement, 50% peripheral arthritis, 25% uveitis and none dactylitis. For 17 patients, there was data on HLA B27 and 76.4% were positive. Regarding treatment, 52.7% were using non-steroidal anti-inflammatory drugs; 11.1% were on methotrexate; 38.8% were using sulphasalazine, 19.4% were on prednisone and 25% were on anti-TNF-alpha drugs (19.4% were on etanercept and 5.5% on adalimumab).

**Figure 1.**
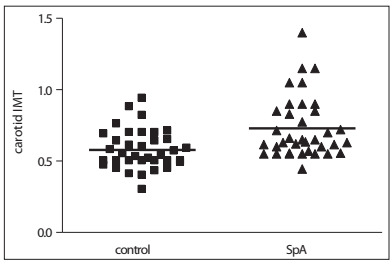
Carotid intima-media thicknesses in 36 patients with spondyloarthritis and 36 controls.

The data relating to sample pairing is shown in Table 1. The carotid IMT in the controls ranged from 0.3 to 0.67 mm (mean 0.57 ± 0.13 mm); in SpA patients, it ranged from 0.44 to 1.40 mm (mean 0.72 ± 0.21 mm), with P = 0.0007 (Figure 1).

**Table 1.** Gender, age, diabetes mellitus and lipid profile among 36 patients with spondyloarthritis and 36 controls

	Controls n = 36	Spondyloarthritis patients n = 36	P
Gender	12 males/24 females	17 males/19 females	0.33
Mean age (years)	42.89 ± 11.70	43.92 ± 10.97	0.70
Diabetes mellitus	1	0	1.00
Arterial hypertension	26.9%	22.2%	0.76
Tobacco exposure	12%	25%	0.27
Mean cholesterol (mg/dl)	179.1 ± 34.06	194.6 ± 31.99	0.10
Mean triglycerides (mg/dl)	145.1 ± 59.12	118.4 ± 63.27	0.13
HDL cholesterol (mg/dl)	39.43 ± 4.664	43.15 ± 10.99	0.14
LDL cholesterol (mg/dl)	126 ± 29.82	115 ± 27.38	0.24

HDL = high-density lipoprotein; LDL = low-density lipoprotein.

SpA = spondyloarthritis; IMT = intima-media thickness.

In the SpA sample, the ESR ranged from 2 to 100 mm (median 13.5 mm); the CRP values were between 0.3 and 4.64 mg/dl (median 0.34 mg/dl). Analysis on carotid IMT according to the functional index and inflammatory activity parameters did not find any difference, as seen in Table 2.

**Table 2.** Correlation of inflammatory disease activity markers and functional index with carotid intima-media thickness in 36 patients with spondyloarthritis

	R	95% CI	P
BASDAI	0.19^*^	- 0.14 to 0.49	0.26
ESR (mm, 1^st^ hour)	0.046^†^	- 0.35 to 0.31	0.89
CRP (mg/dl)	0.046^†^	- 0.29 to 0.37	0.79
BASFI	0.25^*^	- 0.07 to 0.53	0.13

BASDAI = Bath Ankylosing Spondylitis Activity Index; BASFI = Bath Ankylosing Spondylitis Functional Index; ESR = erythrocyte sedimentation rate; CRP = C-reactive protein; CI = confidence interval; ^*^Pearson correlation; ^†^Spearman correlation.

**Figure 2.**
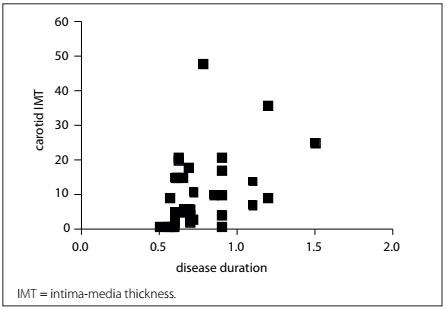
Correlation of carotid intima-media thickness with disease duration in 36 patients with spondyloarthritis.

From studying the carotid IMT according to disease duration, we observed a positive correlation, with P = 0.014 (Pearson R = 0.409; 95% confidence interval, CI = 0.06 to 0.65) (Figure 2). From analysis on the lipid profile and IMT in SpA patients, we did not find any correlation with total cholesterol (P = 0.42), HDL cholesterol (P = 0.39) or LDL cholesterol (P = 0.39). However, there were positive correlations with triglycerides (P = 0.02; Spearman R = 0.37; 95% CI = 0.03 to 0.64) and the patient’s age (P = 0.0014; Pearson R = 0.51; 95% CI = 0.21 to 0.72). From studying carotid IMT in the SpA patients according to SpA subtypes, no differences could be found (P = 0.29). From analyzing carotid IMT according to clinical manifestations, we found the data shown in Table 3.

**Table 3.** Mean values of carotid intima-media thickness (in mm) in patients with and without spondyloarthritis, according to clinical findings

	With	Without	P
Axial involvement	0.75 ± 0.22	0.77 ± 0.28	0.91
Peripheral arthritis	0.76 ± 0.24	0.74 ± 0.20	0.82
Uveitis	0.86 ± 0.32	0.71 ± 0.17	0.12
Entheseal involvement	0.74 ± 0.25	0.77 ± 0.19	0.69
Positive HLA B27 (n = 17)	0.82 ± 0.28	0.66 ± 0.17	0.30

HLA = human leukocyte antigen.

Patients on anti-TNF-alpha drugs had IMT between 0.49 and 1.5 mm (mean 0.8 ± 0.33 mm) and those not using these drugs had IMT between 0.50 and 1.2 mm (mean 0.73 ± 0.18 mm); P = 0.49.

## DISCUSSION

Arterial wall thickening has a strong prognostic value for cardiovascular events, and carotid IMT assessment allows easy identification of patients at risk, as shown in a recent systematic review and meta-analysis.^26^ In particular, it was demonstrated that an absolute difference of 0.1 mm increases the risk of future myocardial infarction by 10 to 15% and the stroke risk by 13 to 18%.^26^^,^^27^ The use of carotid IMT as a predictor of cardiovascular events has predominantly been reported in the literature in relation to patients with rheumatic diseases.^3^

It is well known that in chronic inflammatory rheumatic diseases, systemic inflammation can act independently or synergistically with traditional risk factors in relation to development of cardiovascular complications, but each rheumatic disease may differ regarding the amount and type of inflammation.^27^ In rheumatoid arthritis, the severity of disease has consistently been proven to be the major determinant of myocardial infarction and stroke.^28^ The same has been proven for PsA,^29^ but other types of SpA have not been so well studied. In cardiovascular disease, elevated CRP and interleukin (IL)-6 levels correlate with adverse events.^5^ In rheumatoid arthritis, CRP levels are increased, and this correlates well with disease activity but in SpA, CRP is usually not a good guide for inflammation. It is elevated in only 50-60% of the cases of active disease.^7^ Despite these differences between SpA and rheumatoid arthritis, we found in the present study that SpA patients were also at risk of developing accelerated atherosclerosis.

Our findings confirm those of Gonzalez-Gay et al.,^4^ who studied 64 SpA patients and found that they had greater carotid IMT and carotid plaque than shown by controls. These authors also found an association between this finding and disease duration, as we also did. This finding probably reflects the additive effect of prolonged inflammation.

Although the atherosclerotic process is associated with inflammation, we could not demonstrate any association for carotid IMT in relation to ESR, CRP or BASDAI. As already mentioned, CRP levels are not good markers for disease inflammation in SpA. Peters et al.^30^ also found that carotid IMT did not show any association with CRP or BASDAI in ankylosing spondylitis patients. These findings can be explained by the fact that BASDAI and ESR are measurements that reflect current inflammation while the atherosclerotic process is built up over time. Furthermore, the use of anti-TNF-alpha did not alter the IMT in the present analysis. Even though this drug has well-known anti-inflammatory properties,^31^ its action on the lipid profile is debatable.^32^^,^^33^^,^^34^ An increase in triglyceride levels has been shown in anti-TNF-alpha users with PsA.^32^ in RA cases, some authors^33^ have found an increase in HDL cholesterol, while others have not noted any difference in the atherosclerotic index (total cholesterol/HDL cholesterol).^34^ Modifications to the lipid profile may compensate for the anti-inflammatory effect of this drug.

No special clinical characteristic was associated with higher carotid IMT in SpA patients in the present study. The same has been noted by others.^4^ Thus, surveillance of atherosclerotic and inflammatory processes should also be done for all SpA patients, in order to avoid their severe consequences.

## CONCLUSIONS

Concluding, patients with SpA have higher carotid IMT than shown by controls. Every effort should be made in order to control inflammation and traditional risk factors in this population, to avoid the consequences of accelerated atherogenesis.
